# Discovery of Brominated Alboflavusins With Anti-MRSA Activities

**DOI:** 10.3389/fmicb.2021.641025

**Published:** 2021-02-16

**Authors:** Chao Li, Wuyundalai Bao, Haoyue Zhang, Zhitang Lyu, Yihua Chen, Zhengyan Guo

**Affiliations:** ^1^State Key Laboratory of Microbial Resources, CAS Key Laboratory of Microbial Physiological and Metabolic Engineering, Institute of Microbiology, Chinese Academy of Sciences, Beijing, China; ^2^College of Life Sciences, University of Chinese Academy of Sciences, Beijing, China; ^3^College of Food Science and Engineering, Inner Mongolia Agricultural University, Hohhot, China; ^4^Key Laboratory of Microbial Diversity Research and Application of Hebei Province, Institute of Life Science and Green Development, College of Life Sciences, Hebei University, Baoding, China

**Keywords:** Alboflavusins, structural elucidation, MRSA, antibacterial activity, bromination

## Abstract

As methicillin-resistant *Staphylococcus aureus* (MRSA) is becoming a serious pathogenic threaten to human health worldwide, there is an urgent need to discover new antibiotics for the treatment of MRSA infections. Alboflavusins (AFNs) are a group of halogenated cyclohexapeptides with anti-MRSA activities. In this study, two novel brominated AFN congeners (compounds **1** and **2**) were isolated from the wild-type strain *Streptomyces alboflavus* sp. 313 that was fermented in the production medium supplemented with NaBr; two new (compounds **3** and **5**) and a known (compound **4**) dehelogenated AFN congeners were isolated from *S. alboflavus* Δ*afnX*, in which the tryptophan halogenase gene *afnX* was inactivated. The structures of these compounds were assigned by careful NMR and MS analyses. The anti-MRSA activities of varied AFN congeners were assessed against different MRSA strains, which revealed that compounds **1** and **2** with bromine displayed effective activities against the tested MRSA strains. Especially, compound **2** showed good anti-MRSA activity, while compounds **3**, **4**, and **5** without halogen exhibited weak anti-MRSA activities, outlining the influence of halogen substitution to the bioactivities of AFNs.

## Introduction

*Staphylococcus aureus* (SA) has become a serious pathogenic threat to human health worldwide (Tong et al., 2015; Lee et al., 2018). It usually causes superficial skin and soft tissue infections, sometimes even leading to fatal blood infections and pneumonia (Bhattacharya et al., 2015; Water et al., 2015). In the latest global assessment of antibiotic resistance, SA is considered to be a high priority pathogen (Tacconelli et al., 2018). Specifically, methicillin-resistant SA (MRSA) showed the higher pathogenicity, infection rate and mortality rate (Cosgrove et al., 2003; Stefani et al., 2012). In the United States, the mortality number caused by MRSA is about 19,000 people in 2005, which is higher than the number caused by acquired immune deficiency syndrome (AIDS) (Zouhir et al., 2016). Antibiotic therapy has been considered the primary treatment strategy for SA infection (Bal et al., 2017), and its widespread application has greatly improved the prognosis of patients with SA infection (Aguilar et al., 2010). Presently, vancomycin treatment remains the most important first-line therapy for severe MRSA infection (Okwu et al., 2019). However, the emergence of MRSA with reduced susceptibility to vancomycin (Ghahremani et al., 2018) as well as daptomycin (Roch et al., 2017) and linezolid resistance (de Dios Caballero et al., 2015) has been reported. Given that bacteria naturally evolve toward developing resistance to all antibiotics they are exposed to, it is urgent to search for novel antibacterial agents as well as innovative approaches to combat MRSA (Hoffman and Outterson, 2015; Kaur and Chate, 2015; Ventola, 2015; Aslam et al., 2018).

In our previous study for exploring new antibiotics, alboflavusins (AFNs, which were originally named as NW-Gs), isolated from *Streptomyces alboflavus* sp. 313, were identified as novel cyclic hexapeptide antibiotics with a chlorine atom and exhibited strong antimicrobial activity against many Gram-positive bacteria including MRSA (Guo et al., 2009; Ji et al., 2012a; Wei et al., 2012). It was proposed that an L-tryptophan (Trp) 6-halogenase AfnX is responsible for the halogenation of L-Trp to generate 6-Cl-L-Trp as a precursor of AFN biosynthesis (Guo et al., 2018). Trp halogenases like AfnX are flavin-dependent enzymes usually possessing high region specificity toward the Trp substrate (Buchler et al., 2019), while they may have high substrate promiscuity toward the halide ions and can take Br^–^ and/or I^–^, which can be used for the generation of congeners with different halogen substituents (Latham et al., 2018; Buchler et al., 2019; Lee et al., 2020).

In this study, we replaced NaCl by NaBr or NaI (data not shown) in the fermentation medium and got two novel brominated AFN congeners (**1** and **2**). In addition, by careful analysis of the gene *afnX* inactivated mutant *S. alboflavus* Δ*afnX*, three dechlorinated AFNs were obtained (**3**–**5**) including two new AFN congeners (**3** and **5**). The inhibition activities against several MRSA strains were evaluated for compounds **1**–**5** and AFN A_1_, which clearly showed that halogenated AFNs performed better than the dechlorinated AFNs.

## Materials and Methods

### Strains, Culture, and Fermentation

The wild-type strain *S. alboflavus* sp. 313 was isolated from a soil sample collected in Shaanxi Province, China. *S. alboflavus* Δ*afnX* was a gene in-frame deletion mutant constructed using a CRISPR/Cas9 gene inactivation system in our lab (Guo et al., 2018). The antibacterial activities were performed using five SA strains, including SA ATCC 6538, MRSA 113, MRSA 1.2386, MRSA 09R496, and MRSA 09L098.

*Streptomyces alboflavus* sp. 313 and *S. alboflavus* Δ*afnX* mutant were cultured in plate medium MS (2% soya flour, 2% mannitol, and 2% agar) at 28°C for 7 days. A two-stage culture procedure was used for obtaining AFN congeners. The spore suspension of *S. alboflavus* sp. 313 was inoculated into 250 mL flasks containing 50 mL medium G2 (1% glucose, 0.3% peptone, 0.45% NaBr, 0.1% CaCO_3_, and pH 7.0) as the seed medium at 28°C, 220 r/min for 2 days. Then the seed culture was incubated (1:10, v/v) into 1 L flasks containing 200 mL medium G2 at 28°C, 220 r/min for 5 days.

### Extraction and Isolation of AFNs

After centrifugation, the supernatant of 30 L *S. alboflavus* sp. 313 or *S. alboflavus* Δ*afnX* culture broth was triply extracted with an equal volume of ethyl acetate. The combined extracts were concentrated in vacuum at 35°C. After evaporation, the sample was subjected to a silica gel column (600 g, 200–300 mesh, 6.5 cm × 110 cm) and eluted with petroleum ether, petroleum ether/ethyl acetate (50:50, v/v), ethyl acetate, ethyl acetate/methanol (50:50, v/v), and methanol. Each eluted fraction was concentrated and analyzed by HPLC. The fractions containing AFN congeners were loaded onto a C_18_ flash column (100 μm, 25 mm × 165 mm) and eluted with 200 mL of 20% methanol and 100% methanol. The methanol fractions containing AFN congeners were further fractionated on a Sephadex LH-20 column (3.0 cm × 120 cm) and eluted with methanol. Finally, the fractions containing AFN congeners were concentrated and further purified by semipreparative HPLC (Zorbax SB C_18_, 5 μm, 9.4 mm × 250 mm, Agilent, Santa Clara, CA, United States) eluted with methanol/water (70:30, v/v) containing 0.1% trifluoroacetic acid in 40 min at a flow rate of 3.5 mL/min to get AFN congeners.

### Spectroscopic Analyses

HPLC detection of AFN congeners was performed on a Shimadzu HPLC system (Shimadzu, Kyoto, Japan) by an Apollo C_18_ column (5 μm, 4.6 mm × 250 mm, Alltech, Deerfield, IL, United States). The Apollo C_18_ column was eluted with acetonitrile and water containing 0.1% trifluoroacetic acid at a flow rate of 1.0 mL/min. Percentage of acetonitrile was changed linearly from 15 to 50% in 5 min, from 50 to 87% in 25 min, 87% for 5 min, and from 87 to 100% in 5 min. The detection wavelength was 220 nm. LC-MS analyses were performed on an Agilent 1260/6460 Triple-Quadrupole LC/MS system (Santa Clara, CA, United States) with an electrospray ionization source. HR-ESI-MS was performed on an Agilent 1260 HPLC/6520 QTOF-MS instrument (Santa Clara, CA, United States). NMR spectra were recorded at room temperature on a Bruker-500 NMR spectrometer (Billerica, MA, United States).

### Minimum Inhibitory Concentration Testing

According to the protocols of the Standard of National Committee for Clinical Laboratory, the antibacterial activities of AFN congeners were measured by the microbroth dilution method in 96-well culture plates using Mueller-Hinton broth (Qingdao Nissui Biotechnologies Co., Ltd., Shandong, China) (Wiegand et al., 2008). Briefly, the tested bacteria were cultured in Mueller-Hinton agar plates at 37°C for 12 h. Then the single colonies of tested bacteria were incubated in Mueller-Hinton broth at 37°C, 220 r/min for 6 h. Then the cell concentrations were diluted to approximately 1 × 10^6^ colony-forming units (CFU) with Mueller-Hinton broth. Each of the tested compounds was dissolved in DMSO and then diluted with sterile water by the twofold dilution method. The final concentrations of each sample in 96-well culture plates were 200, 100, 50, 25, 12.50, 6.25, 3.13, 1.56, 0.78, 0.39, and 0.20 μM. Vancomycin and daptomycin were used as positive controls. Mueller-Hinton broth was used as a negative control. The tested bacteria were then incubated at 37°C for 18 h and the minimum inhibitory concentration (MIC) values were calculated.

## Results

### Discovery of Two Brominated and Three Dechlorinated AFN Congeners From *Streptomyces alboflavus* sp. *313 and*Δ*afnX, Respectively*

**FIGURE 1:**
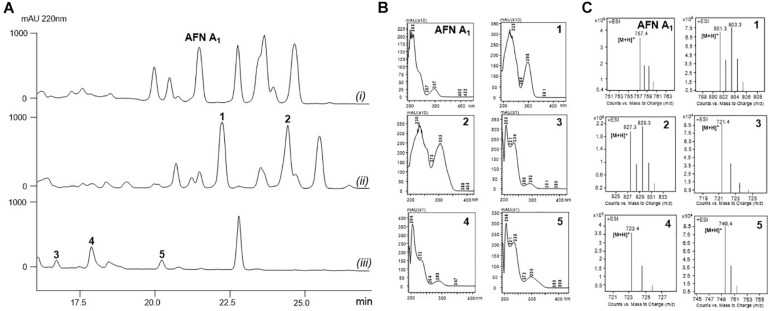
Metabolic profiles of *S. alboflavus* sp. 313 and the Δ*afnX* mutant. **(A)** HPLC traces of the supernatant extracts of *S. alboflavus* sp. 313 and the Δ*afnX* mutant at 220 nm. *S. alboflavus* sp. 313 fermented in medium G2 with (i) NaCl and (ii) NaBr; (iii) *S. alboflavus* Δ*afnX* mutant fermented in medium G2 with NaBr. **(B)** UV absorptions of AFN A_1_ and **1**–**5**. **(C)** MS analyses of AFN A_1_ and **1**–**5**.

The fermentation broth of *S. alboflavus* sp. 313 or *S*. *alboflavus* Δ*afnX* was harvested, extracted, and analyzed by LC-MS. According to the similarity of UV adsorption and the isotope abundance peaks (Figure 1), two major AFN congeners (**1** and **2**) containing one bromine atom were discovered from *S. alboflavus* sp. 313, while three AFN congeners without chlorine (**3**, **4**, and **5**) were detected in the crude extract of *S. alboflavus* Δ*afnX*.

Actually, no AFN congener with bromine has been described before. For dechlorinated AFNs, only one compound, AFN A_2_, was discovered from *S*. *alboflavus* sp. 313 (Fan et al., 2013). Therefore, compounds **1**–**5** have a good chance to be novel AFNs. Therefore, 30 L fermentation broths of *S. alboflavus* sp. 313 and the Δ*afnX* mutant were collected for compound isolation and finally 10.2 mg **1**, 6.7 mg **2**, 3.4 mg **3,** 5.6 mg **4**, and 4.2 mg **5** were obtained for structure elucidation.

### Structural Elucidation of Compounds 1 and 2 With Bromide Substitution

**FIGURE 2:**
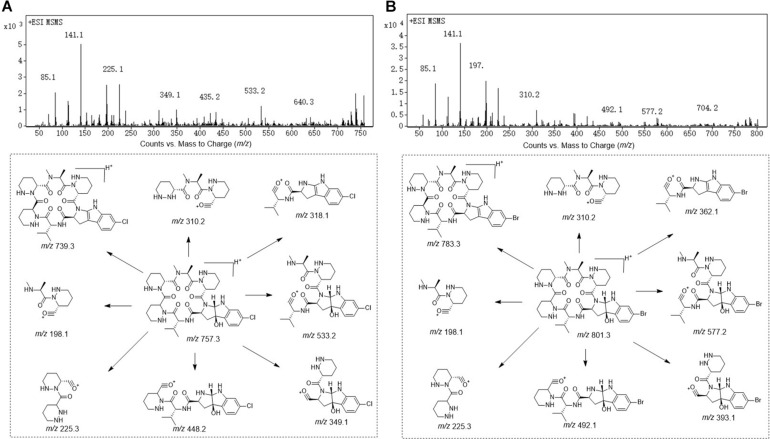
The MS/MS spectra of the known AFN A_1_ **(A)** and **1 (B)**.

**FIGURE 3:**
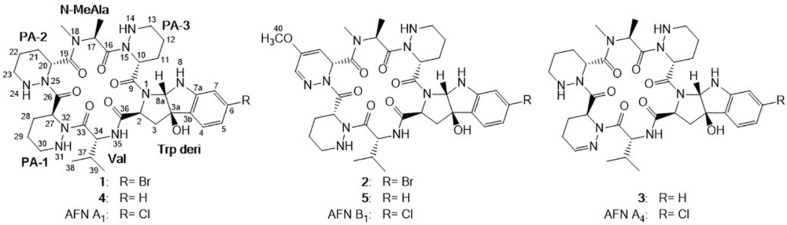
Structures of AFN congeners A_1_, A_4_, B_1_, and **1**–**5**.

Compound **1** was obtained as white powder with the molecular formula C_35_H_49_BrN_10_O_7_ based on the presence of an ion in its HR-ESI-MS at *m/z* 801.3071 [M + H]^+^ [calcd: 801.3042, (M + H)^+^]. The presence of one bromine atom was suggested by the isotope abundance peaks in the MS spectrum. The^1^H and ^13^C NMR of **1** (Table 1 and Supplementary Figure 1) are extremely similar to those of AFN A_1_ (also named NW-G01). In ^1^H NMR, the signals of the aromatic protons δ_H_ 7.17 (d, *J* = 8.0 Hz), δ_H_ 6.96 (dd, *J* = 8.0, 1.5 Hz), and δ_H_ 6.85, (d, *J* = 1.5 Hz) were assigned as H-4, H-5, and H-7 of the indol aromatic ring by comparison with the corresponding chemical shifts and coupling constants of AFN A_1_, illustrating that **1** was brominated at C-6 position. These data suggested that **1** can have the same structure of AFN A_1_ where the chlorine atom in the latter has been replace by a bromine in **1**. To verify the structure assignment, MS/MS analyses of AFN A_1_ and **1** were performed (Figure 2). As anticipated, compound **1** fragment ions (*m/z* 783, 577, 492, 393, and 362) including a bromine atom were 44 mass units more than AFN A_1_ fragment ions (*m/z* 739, 533, 448, 349, and 318) including a chlorine atom. In addition, **1** and AFN A_1_ contained the same fragmentation ions (*m/z* 310, 225, and 198). These results confirmed that **1** has the same structure as AFN A_1_ except that **1** has a C-6 bromine atom (Figure 3).

**TABLE 1:** ^1^H and ^13^C NMR data of compounds **1** and **2** (δ_H_ in ppm, *J* in Hz).

No.	1^a^		2^a^			1^a^		2^a^	
	δ_C_	δ_H_, mult. (*J* in Hz)	δ_C_	δ_H_, mult. (*J* in Hz)	No.	δ_C_	δ_H_, mult. (*J* in Hz)	δ_C_	δ_H_, mult. (*J* in Hz)
2	61.6	5.16, d (8.5)	61.6	5.12, d (8.5)	19	169.9		169.1	
3	39.1	2.71, d (14.0); 2.04, dd (14.0, 8.5)	39.2	2.72, d (15.5); 2.11, dd (14.0, 8.5)	20	52.1	5.38, brd (3.5)	53.5	5.98, d (6.5)
3a	89.9		89.8		21	25.3	2.30, brd (14.5); 1.72, m	93.1	5.18, d (6.5)
3b	130.4		130.4		22	19.9	2.39, m; 1.47, m	146.3	
4	124.2	7.17, d (8.0)	124.3	7.17, d (8.0)	23	47.9	3.13, m; 2.76, m	135.9	6.84, s
5	123.0	6.96, dd (8.0, 1.5)	123.1	6.96, dd (8.0, 2.0)	26	172.5		170.4	
6	123.1		123.2		27	43.9	5.33, m	44.2	5.22, t (4.0)
7	114.5	6.85, d (1.5)	114.6	6.81, d (3.0)	28	24.3	2.80, m; 1.86, m	24.0	2.79, m; 1.84, m
7a	148.5		148.6		29	19.3	2.39, m; 1.47, m	19.4	2.32, m; 1.50, m
8a	86.2	5.19, s	85.9	5.19, s	30	46.6	3.13, m; 2.72, m	47.7	3.19, m; 2.75, m
9	170.2		170.3		33	175.5		174.5	
10	50.5	5.30, dd (6.5, 2.5)	52.1	5.39, d (5.0)	34	54.1	5.44, dd (10.0, 7.5)	54.8	5.52, m
11	25.0	2.17, m; 1.86, m	25.3	2.29, m; 1.74, m	35-NH		7.69, d (10.0)		7.71, d (10.0)
12	21.8	1.60, m; 1.47, m	19.9	2.32, m; 1.50, m	36	172.2		172.3	
13	48.3	3.04, dd (14.0, 4.5); 2.82, d (3.0)	48.2	3.02, dd (13.0, 4.0); 2.79, m	37	30.2	2.00, m	29.8	2.16, m
16	174.6		174.4		38	18.4	0.99, d (7.0), 3H	17.3	1.00, d (7.0), 3H
17	49.9	5.62, q (7.0)	49.7	5.63, q (7.0)	39	19.6	0.97, d (7.0), 3H	19.7	0.97, d (7.0), 3H
17-CH_3_	14.5	1.23, d (7.0), 3H	14.5	1.24, d (7.0), 3H	40			54.8	3.61, s, 3H
N_18_-CH_3_	31.0	2.87, s, 3H	31.0	2.88, s, 3H					

*^*a*^Spectra of compounds **1** and **2** were recorded at ^1^H NMR (500 MHz), and ^13^C NMR (125 MHz) with CDCl_3_ as solvent.*

Compound **2** was obtained as gray powder with the molecular formula C_36_H_47_BrN_10_O_8_ based on HR-ESI-MS at *m/z* 827.2836 (M + H)^+^ [calcd: 827.2834, (M + H)^+^]. Its MS analysis also suggested the presence of one bromine atom by the isotope abundance peaks. The^1^H and ^13^C NMR data of **2** (Table 1 and Supplementary Figure 2) were extremely similar to those of AFN B_1_ (also named NW-G06) (Ji et al., 2012b). In ^1^H NMR, there were three signals of the aromatic protons δ_H_ 7.17 (d, *J* = 8.0 Hz; H-4), δ_H_ 6.96 (dd, *J* = 8.0, 2.0 Hz; H-5), and δ_H_ 6.81 (d, *J* = 3.0 Hz; H-7), suggesting that **2** was brominated at C-6 position. Besides, there were the presence of two *sp*^2^ methine protons δ_H_ 5.18 (H-21) and δ_H_ 6.84 (H-23), and the presence of a –OCH_3_ group indicated by the singlet peak at δ_H_ 3.61. Therefore, we proposed that **2** has the same structure as AFN B_1_ where bromine substitutes the C-6 chlorine. To further verify this, MS/MS analyses were conducted for AFN B_1_ and **2** comparably (Supplementary Figure 3B). Compound **2** fragmentation ions (*m/z* 809, 577, 492, 393, and 362) with a bromine atom were 44 mass units more than AFN B_1_ fragment ions (*m/z* 765, 533, 448, 349, and 318) with a chlorine atom. Besides, AFN B_1_ and **2** shared the same fragment ions (*m/z* 336, 251, and 198) without one halogen atom confirming that they share a part of the same structure. In addition, comparison of the fragmentation patterns of **2** and **1** also supported the structure assignment of **2** (Figure 3).

### Structural Elucidation of Compounds 3, 4, and 5 Without Halogen Atom

Compound **3** was obtained as faint yellow powder with the molecular formula C_35_H_48_N_10_O_7_ based on HR-ESI-MS at *m/z* 721.3785 (M + H)^+^ [calcd: 721.3780, (M + H)^+^], and the isotope abundance peaks in the MS spectrum of **3** implied that it has no halogen atom. Compound **3** has the same molecular formula as AFN A_4_ (or NW-G03) (Guo et al., 2011). The^1^H and ^13^C NMR of **3** are summarized in Table 2 (Supplementary Figure 4). They are extremely similar to those of the reported AFN A_2_ (or NW-G12) (Fan et al., 2013) except for the presence of one additional *sp*^2^ proton (δ_H_ 6.93). In ^1^H NMR, there were four aromatic proton signals δ_H_ 7.21 (d, *J* = 7.5 Hz; H-4), δ_H_ 6.72 (t, *J* = 7.5 Hz; H-5), δ_H_ 7.10 (t, *J* = 7.5 Hz; H-6), and δ_H_ 6.64 (d, 7.5 Hz; H-7), indicating that compound **3** lacks the C-6 halogen substitution. In addition, comparative analyses of the MS/MS spectra of **3** (Supplementary Figure 5A) and AFN A_1_ (Figure 2A) showed that compound **3** structure is identical to AFN A_4_ structure expect that **3** has no chlorine atom (Figure 3).

**TABLE 2:** ^1^H and ^13^C NMR data of compounds **3** and **5** (δ_H_ in ppm, *J* in Hz).

No.	3^b^	5^b^			3^b^	5^b^	
	δ_H_, mult. (*J* in Hz)	δ_C_	δ_H_, mult. (*J* in Hz)	No.	δ_H_, mult. (*J* in Hz)	δ_C_	δ_H_, mult. (*J* in Hz)
2	4.43, m	61.2	4.41, m	19		172.0	
3	2.23, m; 2.08, m	41.3	2.22, m; 2.13, m	20	5.70, m	53.7	5.21, dd (6.0, 2.5)
3a		87.0		21	1.91, m; 1.83, m	93.2	6.13, d (6.0)
3b		130.9		22	1.45, m, 2H	145.6	
4	7.21, d (7.5)	123.0	7.22, d (7.5)	23	3.10, brd (12.5); 2.77, d (4.5)	134.9	6.86, d (2.5)
5	6.72, t (7.5)	118.6	6.72, t (7.5)	26		170.4	
6	7.10, t (7.5)	129.2	7.10, t (7.5)	27	5.10, m	45.6	5.38, brd (5.5)
7	6.64, d (7.5)	113.1	6.65, d (7.5)	28	1.96, m, 2H	22.5	1.79, m; 1.98, m
7a		148.1		29	2.03, m, 2H	19.4	1.64, m, 2H
8a	5.34, brs	83.6	5.15, s	30	6.93, brs	47.4	2.97, m, 2H
9		173.7		33		172.3	
10	5.20, brs	53.1	5.13, m	34	5.03, brs	55.0	3.59, brs
11	2.27, m; 1.66, m	26.1	1.79, m; 2.15, m	35-NH			
12	2.06, m, 2H	24.7	1.98, m, 2H	36		169.9	
13	3.00, m; 2.77, d (4.5)	45.9	2.97, m; 2.78, m	37	1.91, m	29.8	1.98, m
16		171.2		38	0.86, d (7.0), 3H	16.8	0.85, m, 3H
17	5.60, q (6.5)	47.6	5.62, q (6.5)	39	0.86, d (7.0), 3H	19.7	0.92, m, 3H
17-CH_3_	1.22, d (7.0), 3H	14.4	1.20, d (7.0), 3H	40		54.7	3.57, s, 3H
N_18_-CH_3_	2.83, s, 3H	30.1	2.85, s, 3H				

*^*b*^Only the ^1^H NMR (500 MHz) spectrum was recorded for **3**. The spectra of **5** were recorded at ^1^H NMR (500 MHz) and ^13^C NMR (125 MHz) with DMSO-_*d6*_ as solvent.*

Compound **4** was also a faint yellow powder. The molecular formula of **4** was determined to be C_35_H_50_N_10_O_7_ based on HR-ESI-MS at *m/z* 723.3938 [M + H]^+^ [calcd: 723.3937, (M + H)^+^], the same as that of AFN A_2_ (Figure 3). The MS spectrum of **4** suggested that it has no halogen atom by the isotope abundance peaks. Compound **4** was confirmed to be AFN A_2_ by the fact that it has the same HPLC retention time as standard AFN A_2_.

Compound **5** was obtained as reddish powder with the molecular formula C_36_H_48_N_10_O_8_ based on HR-ESI-MS at *m/z* 749.3733 [M + H]^+^ [calcd: 749.3729, (M + H)^+^]. No halogen atom specific isotope abundance peak was detected in its MS analysis either. The^1^H and ^13^C NMR data of **5** are summarized in Table 2 (Supplementary Figure 6). Compound **5** was assigned to be the analog of AFN B_1_ (also named NW-G06) (Ji et al., 2012b) without the C-6 halogen substitution. It was supported by the presence of four aromatic proton signals δ_H_ 7.22 (d, *J* = 7.5 Hz; H-4), δ_H_ 6.72 (t, *J* = 7.5 Hz; H-5), δ_H_ 7.10 (t, *J* = 7.5 Hz; H-6), and δ_H_ 6.65 (d, 7.5 Hz; H-7) in its ^1^H NMR. The presence of a –OCH_3_ group was supported by the observation of one singlet peak at δ_H_ 3.57. In addition, the MS/MS spectra comparison of compound **5** and AFN B_1_ verified **5** as dechlorinated AFN B_1_ (Figure 3 and Supplementary Figure 5B).

### Antibacterial Activity of **1*–*5** Against Different MRSA Strains

Our previous study revealed that AFNs with a chlorine atom exhibited promising antibacterial activities against Gram-positive bacteria *S. aureus* including MRSA (Guo et al., 2009; Ji et al., 2012b; Wei et al., 2012). To study the inhibition activities of the **1**–**5** against *S. aureus*, one *S. aureus* strain and four different MRSA strains from the clinic samples were tested. As shown in Table 3, **1** and **2** with a bromine substitution could inhibit different MRSA strains with MIC values ranging from 3.13 to 25 μM. Especially, **2** displayed higher anti-MRSA activities than **1** and the positive control, AFN A_1_. Compounds **4** and **5** showed weak activities against the tested MRSA strains with MIC values ranging from 25 to 50 μM, whereas **3** showed no anti-MRSA activities at 200 μM. The structure activity relationship studies of these AFN congeners indicated that (i) the presence of halogen atoms (chlorine or bromine) can improve the anti-MRSA activity of AFNs; (ii) the unsaturated PA-1 moiety decreases the anti-MRSA activity of **3** significantly; (iii) desaturation and methoxylation of the PA-2 moiety improve the anti-MRSA activity of AFNs.

**TABLE 3:** Antibacterial activities of compounds **1**–**5** (MIC, μM, *n* = 3).

Compounds	MIC values (μM)				
	SA	MRSA1	MRSA2	MRSA3	MRSA4
AFN A_1_	12.5	25	25	25	12.5
1	12.5	25	25	12.5	12.5
2	6.25	3.13	6.25	6.25	3.13
3	>200	>200	>200	>200	>200
AFN A_2_ (4)	50	50	50	50	50
5	25	50	50	50	25
Van	0.39	0.39	0.78	0.39	0.39
Dap	0.39	0.78	0.78	0.78	1.56

*SA, *Staphylococcus aureus* ATCC 6538; MRSA1, 2, 3, and 4, methicillin-resistant *Staphylococcus aureus* strains 113, 1.2386, 09L098, and 09R496; Van, vancomycin; Dap, daptomycin.*

## Discussion

Halogen atoms are frequently observed in pharmaceuticals and agrochemicals, partly on account of the profound effects of halogen substitution on their biological activities (Hernandes et al., 2010; Jiang et al., 2016; Latham et al., 2018). Diverse chemical halogenation methods have been developed, which significantly prompts the halogenated pharmaceutical preparations. However, chemical halogenation methods usually rely on harsh reaction conditions, need multiple protecting and activating steps, and cannot avoid the use of toxic compounds (e.g., halogen gas and Lewis catalysts) (Timmins and de Visser, 2018; Widmann et al., 2020). The development of biocatalytic halogenations serves an alternative and green choice (Mitchell et al., 2017; Schnepel and Sewald, 2017).

Trp halogenases, as a group of promising halogenations biocatalysts, can conduct regio-specifically chlorination at various positions of tryptophan (Wang et al., 2017). Interestingly, several Trp halogenases can also brominate native substrates when a high concentration of bromine is added to the culture medium (Thapa et al., 2018). The Trp 6-halogenase ThaI from Streptomyces *albogriseolus* MJ286-76F7 (Milbredt et al., 2014), SttH from *Streptomyces toxytricini* NRRL 15443 (Zeng and Zhan, 2011), and SatH from *Streptomyces albus* (Lee et al., 2020) can accept both Cl^–^ and Br^–^ as halogen donors to generate 6-halogenated Trp. The discovery of two brominated AFN congeners **1** and **2** suggested that not only the Trp halogenase, AfnX, can use Br^–^ as a halogen donor to generate 6-brominated Trp, but also the whole AFN biosynthetic machinery, including the non-ribosomal peptide synthetase assembly line and the following tailoring enzymes, can tolerate the replacement of 6-Cl Trp to 6-Br Trp. The significant promiscuity of the AFN biosynthetic machinery toward Trp analogs was further outlined by the *S. alboflavus* Δ*afnX* mutant, in which the Trp halogenase gene *afnX* was inactivated. Without 6-halogenated Trp supply in *S. alboflavus* Δ*afnX*, the AFN biosynthetic machinery could take Trp as a substrate to synthesize compounds **3**, **4**, and **5** without halogen atom. Overall, these results suggested that the substrate promiscuities of Trp halogenase and the natural product biosynthetic machinery could be used for convenient and green biosynthesis of natural product congeners with diverse halogenation status.

Alboflavusins were discovered as a group of cyclic chlorinated hexapeptide antibiotics with effective inhibition activities against MRSA strains (Guo et al., 2009; Ji et al., 2012a). It was noticed that chlorination is important to antibacterial activity of AFNs (Fan et al., 2013). The relatively weaker anti-MRSA activities of compounds **3**, **4**, and **5** also outline the importance of halogenations to the bioactivity of AFNs. However, since brominated AFN has never been generated before, the influence on AFN bioactivity of replacing Cl^–^ with Br^–^ remains a mystery. We showed in this work that compound **1** displayed comparable anti-MASR activity as AFN_1_, indicating that, in the case of AFNs, there is no significant difference between bromination and chlorination. Particularly, compound **2** outperformed in the anti-MRSA tests, implying that desaturation and methoxylation of PA-2 moiety can increase the anti-MRSA activity of AFNs. These results laid a solid stage for the following development of AFNs analogs with better anti-MRSA activity.

## Conclusion

In this study, four new AFN congeners **1**, **2**, **3**, and **5** were discovered from *S. alboflavus* sp. 313 and S. *alboflavus* Δ*afnX* mutant. Compounds **1** and **2** were the first report of AFNs congeners with bromine; compounds **3** and **5** were determined to be the dechlorination analogs of AFN A_4_ and AFN B_1_, respectively. Anti-MRSA assays revealed that compounds **1** and **2** with bromine showed antibacterial activities against the tested MRSA strains as promising as that of AFN A_1_. Compounds **4** and **5** displayed weak activities with MIC values of 25–50 μM, whereas **3** showed no activity (MIC > 200 μM). Those results revealed that halogen substitution is important to AFNs for their anti-MRSA activities.

## Data Availability Statement

The original contributions presented in the study are included in the article/Supplementary Material. Further inquiries can be directed to the corresponding author/s.

## Author Contributions

ZG, WB, and YC designed the experiments. ZG, CL, and HZ conducted most of the experiments. ZG, YC, CL, ZL, and WB wrote and edited the manuscript. All authors read and approved the manuscript.

## Conflict of Interest

The authors declare that the research was conducted in the absence of any commercial or financial relationships that could be construed as a potential conflict of interest.
