# Supplementary material for: Discovery of Brominated Alboflavusins With Anti-MRSA Activities
Source: Front Microbiol. 2021 Feb 16;12:641025. doi: 10.3389/fmicb.2021.641025 (PMC7920986; doi:10.3389/fmicb.2021.641025)
Supplement: Supplementary file 1 [file Data_Sheet_1.pdf]

Supplementary Material

Discovery of Brominated Alboflavusins With Anti-MRSA Activities

Chao Li[1,2†], Wuyundalai Bao[3†], Haoyue Zhang[4], Zhitang Lyu[4], Yihua Chen[1,2], Zhengyan Guo[1,2*]

[1]State Key Laboratory of Microbial Resources & CAS Key Laboratory of Microbial Physiological and Metabolic Engineering, Institute of Microbiology, Chinese Academy of Sciences, 100101 Beijing, China

[2]College of Life Sciences, University of Chinese Academy of Sciences, 100049 Beijing, China

[3]College of Food Science and Engineering, Inner Mongolia Agricultural University, 010018 Hohhot, Inner Mongolia, China

[4]Key Laboratory of Microbial Diversity Research and Application of Hebei Province, Institute of Life Science and Green Development, College of Life Sciences, Hebei University, 071002 Baoding, Hebei, China

*** Correspondence:**
Corresponding Author: Zhengyan Guo guozhengyan@im.ac.cn

[†]These authors have contributed equally to this work

Contents

Supplementary Figure S1. Spectral data of **1**. S3

Supplementary Figure S2. Spectral data of **2**. S4

Supplementary Figure S3. The MS/MS spectra of AFN B_1 (A) and **2** (B). S5

Supplementary Figure S4. Spectral data of **3**. S6

Supplementary Figure S5. The MS/MS spectra of **3** (A) and **5** (B). S7

Supplementary Figure S6. Spectral data of **5**. S8

Supplementary Figure S6. Spectral data of **5**. S9

Supplementary Figure S6. Spectral data of **5**. S10

Supplementary Figure S6. Spectral data of **5**. S11

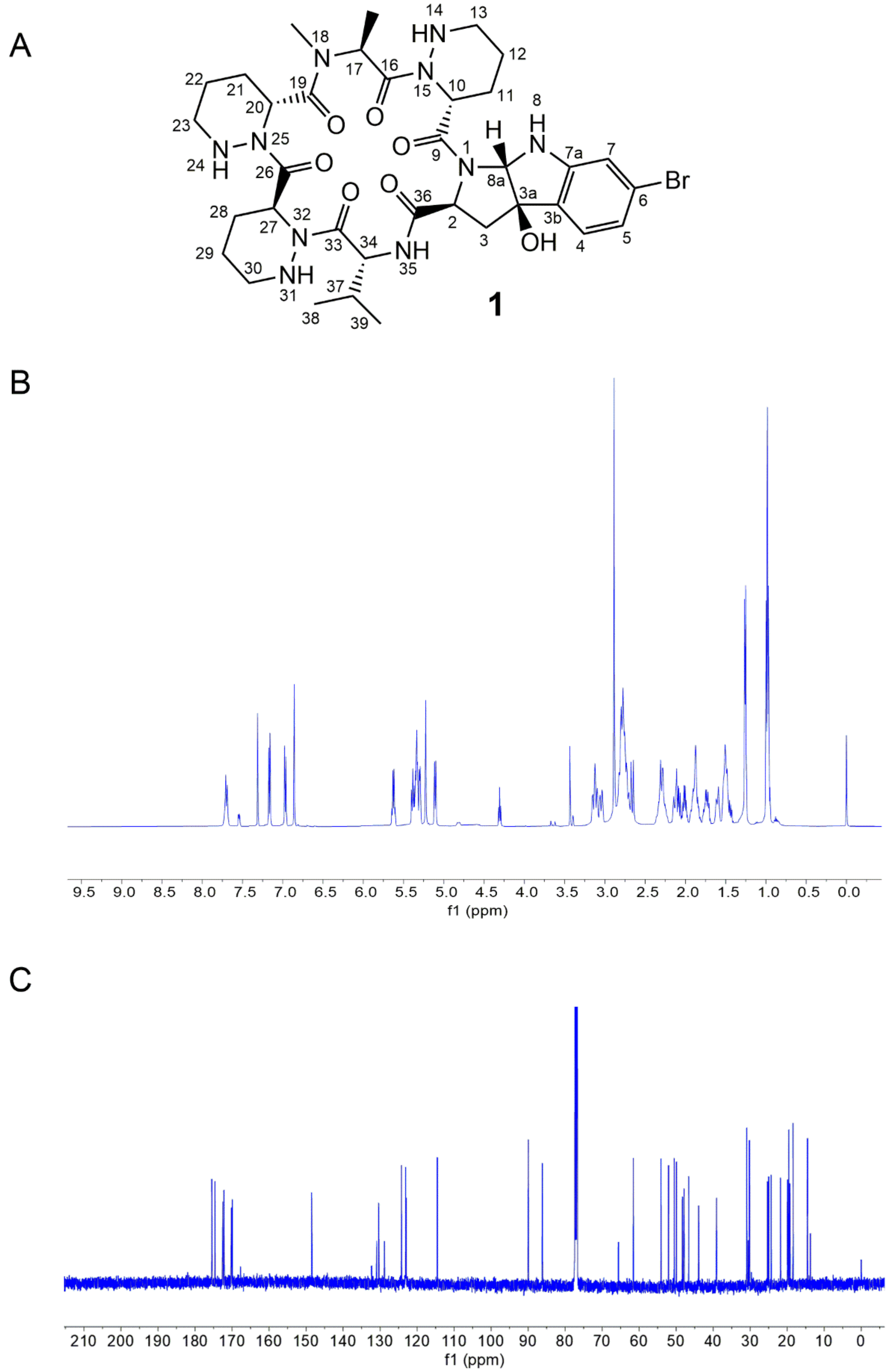

Supplementary Figure S1. Spectral data of **1**. (A) Structure of **1**. (B) ^{1}H NMR spectrum of **1** in $CDCl_3$. (C) ^{13}C NMR spectrum of **1** in $CDCl_3$.

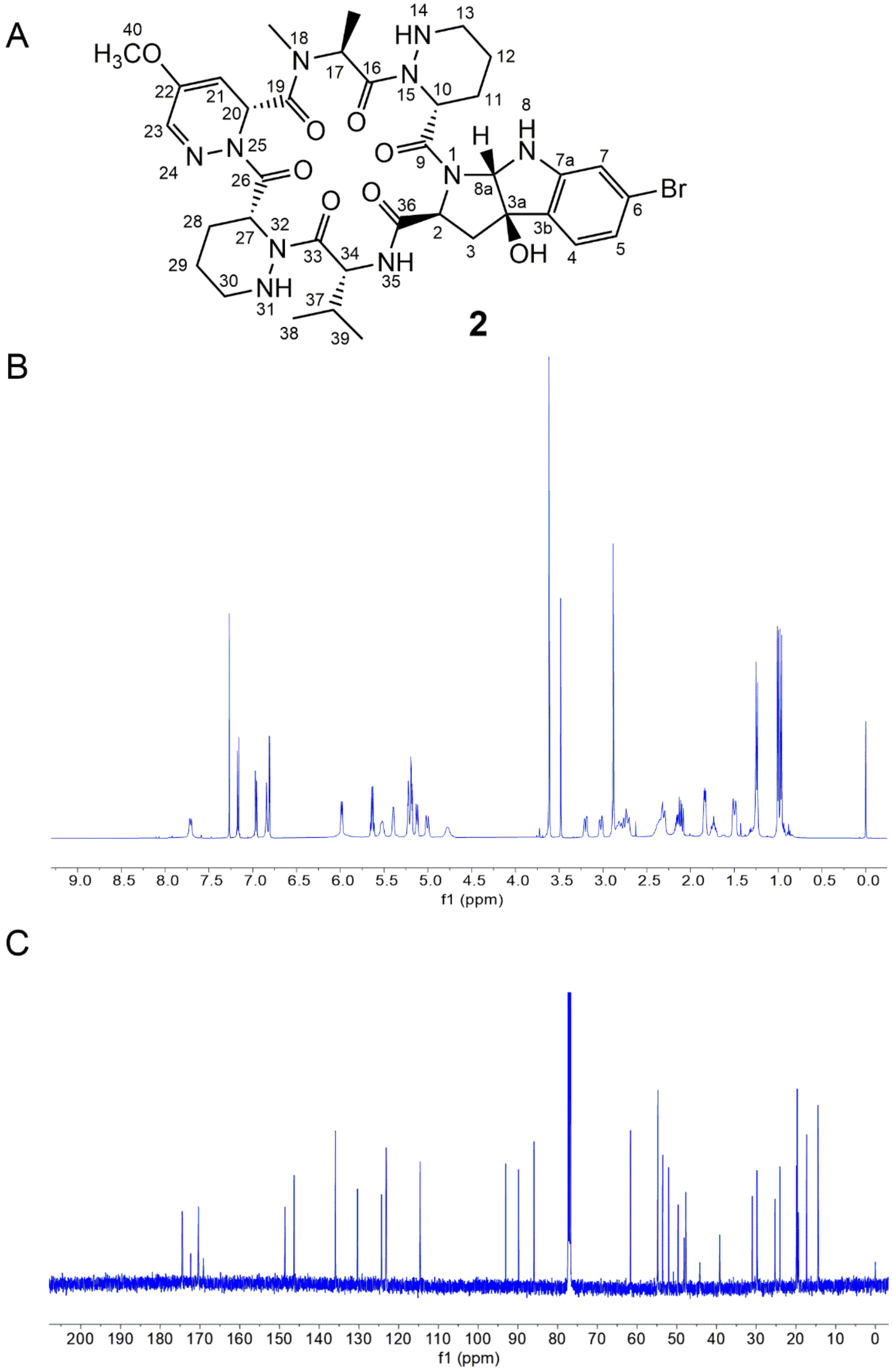

Supplementary Figure S2. Spectral data of **2**. (A) Structure of **2**. (B) ^{1}H NMR spectrum of **2** in $CDCl_3$. (C) ^{13}C NMR spectrum of **2** in $CDCl_3$.

A

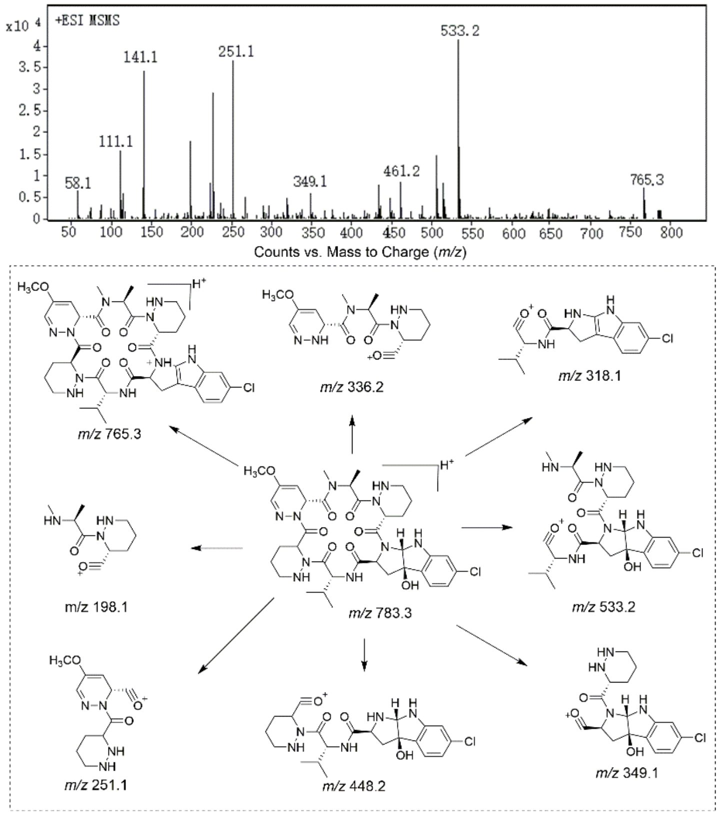

B

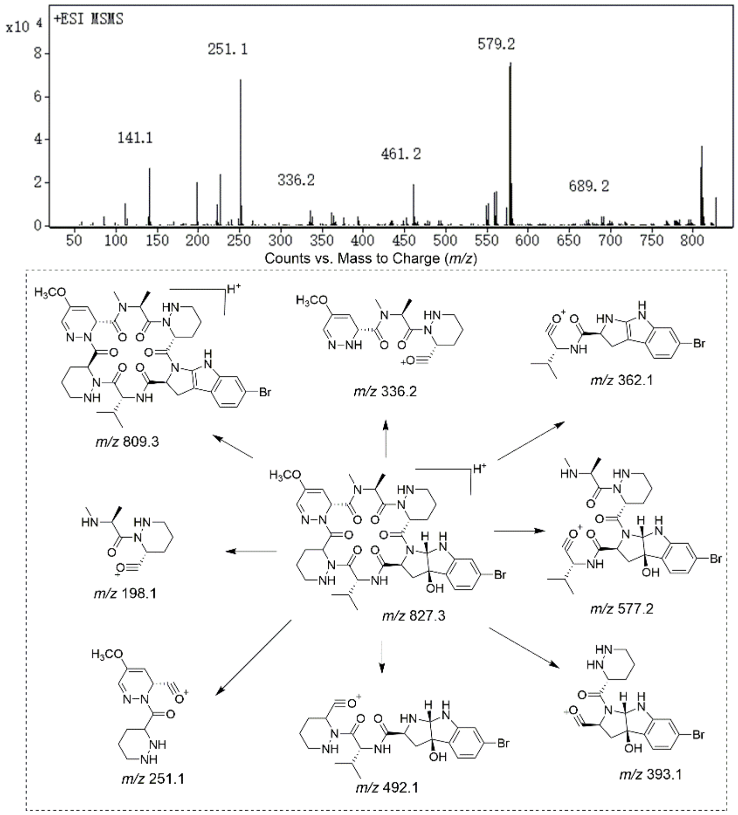

Supplementary Figure S3. The MS/MS spectra of AFN B_1 (A) and **2** (B).

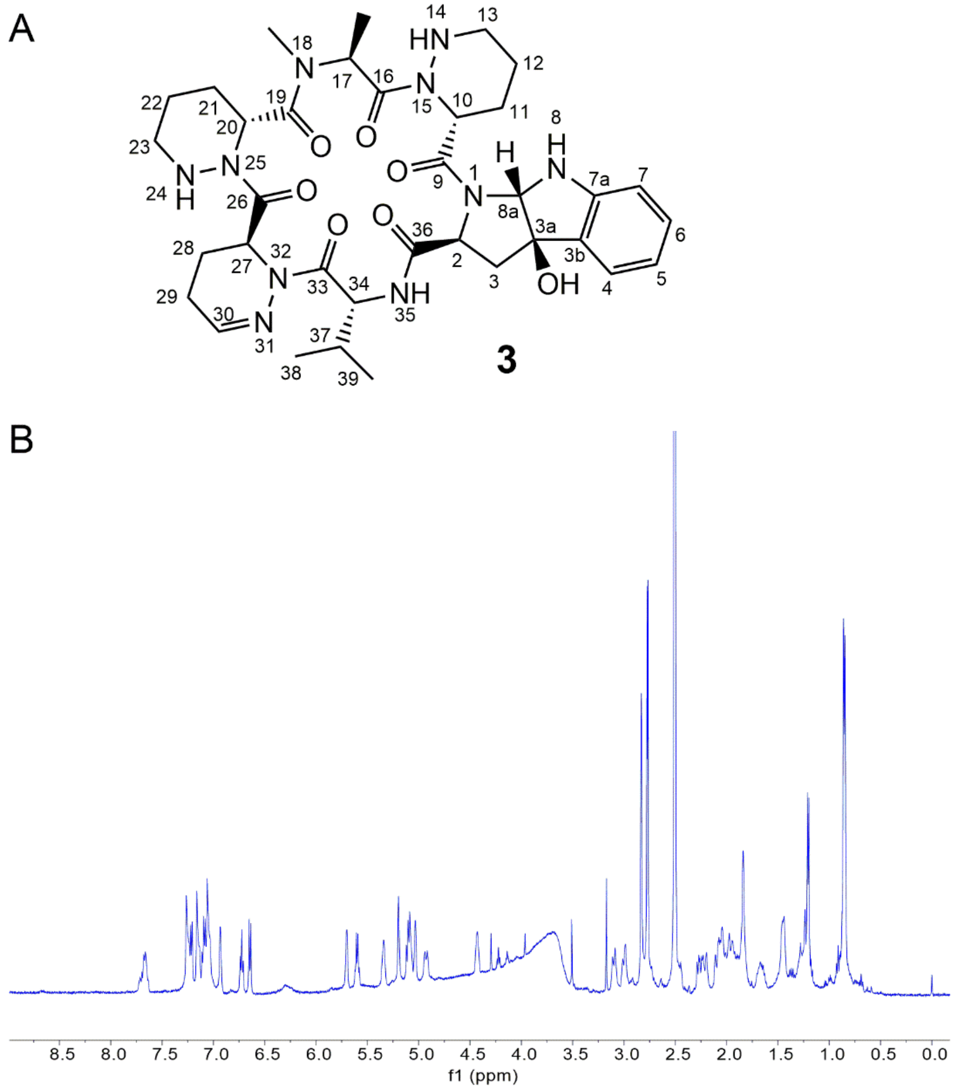

Supplementary Figure S4. Spectral data of **3**. (A) Structure of **3**. (B) ^{1}H NMR spectrum of **3** in DMSO-d_6.

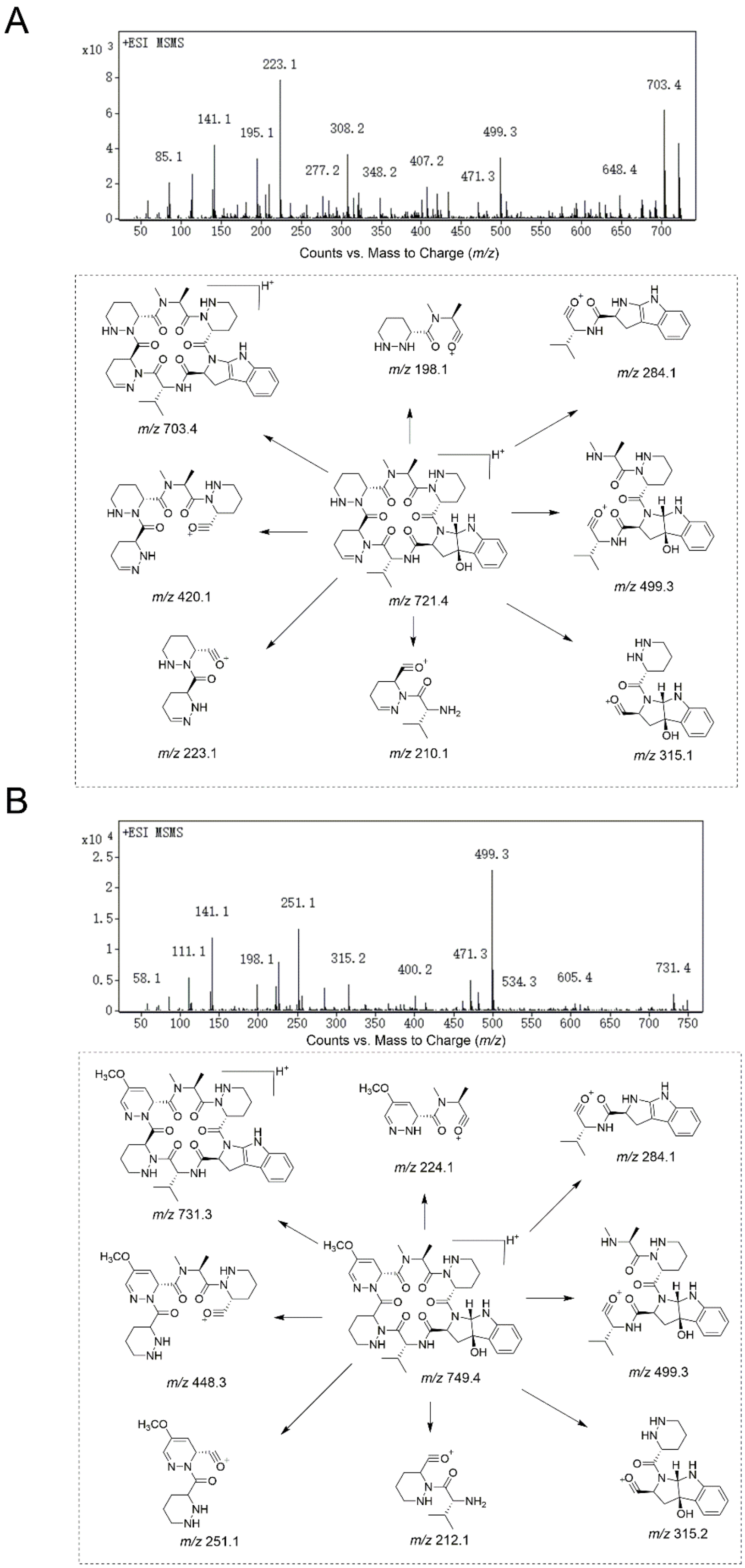

Supplementary Figure S5. The MS/MS spectra of **3** (A) and **5** (B).

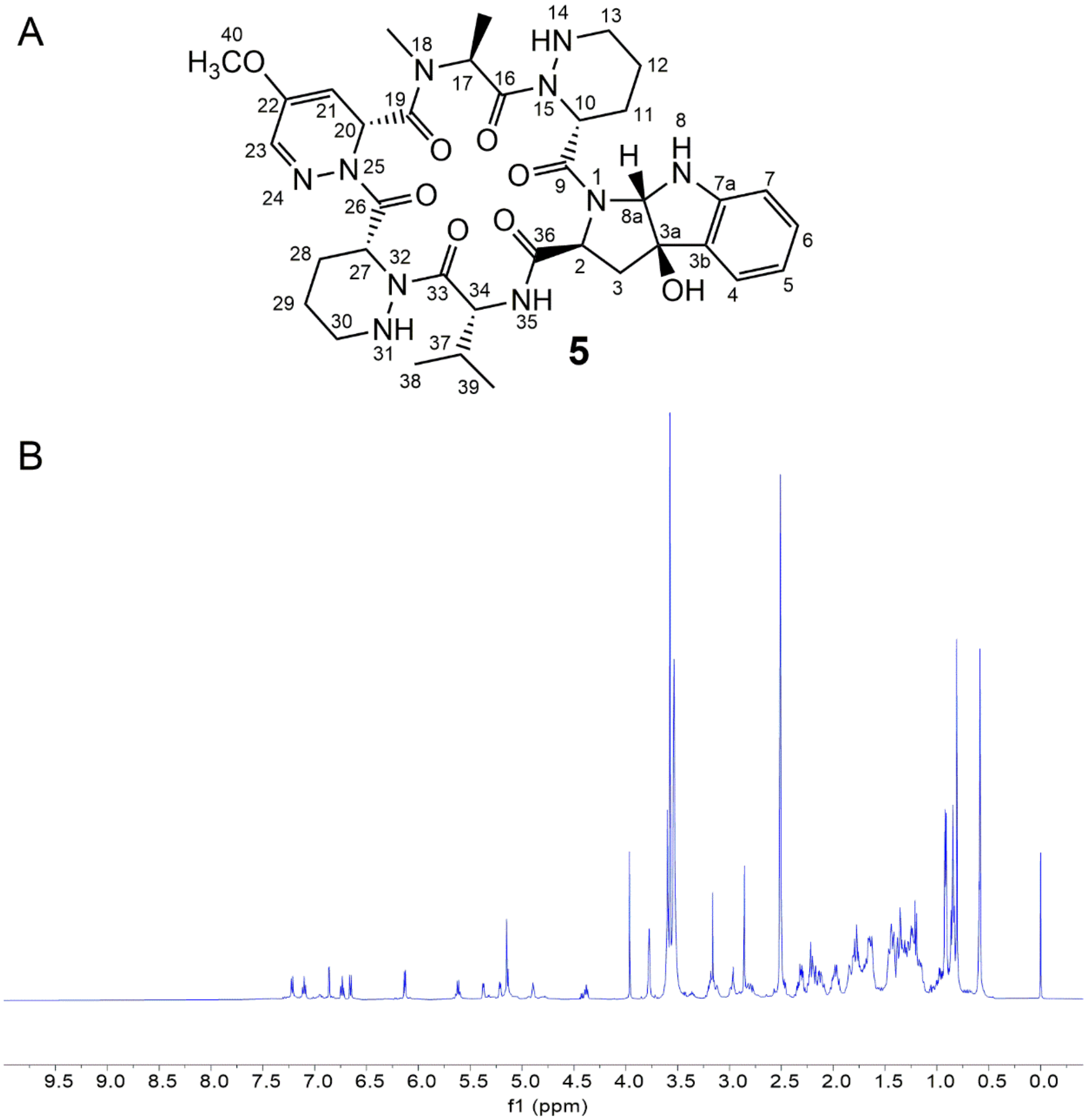

Supplementary Figure S6. Spectral data of **5**. (A) Structure of **5**. (B) ^{1}H NMR spectrum of **5** in DMSO-$_{d6}$.

C

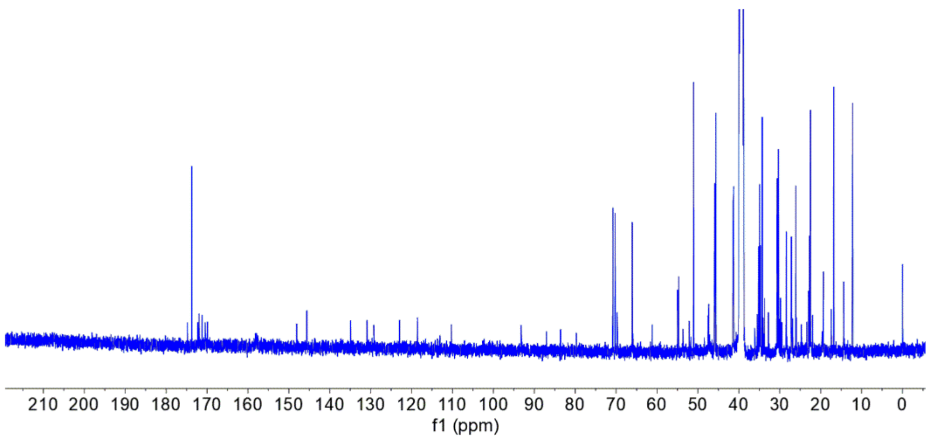

D

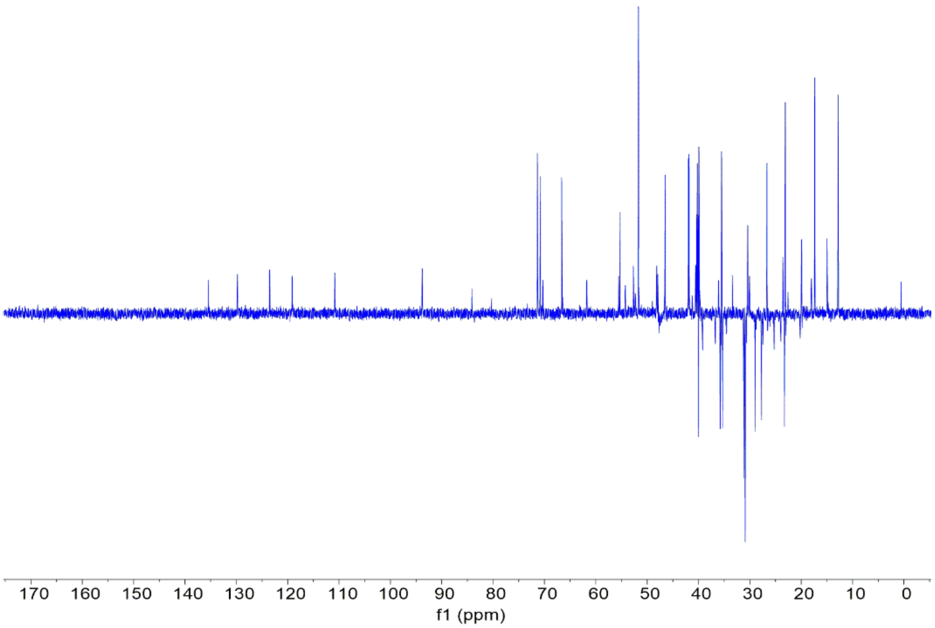

Supplementary Figure S6. Spectral data of **5**. (C) ^{13}C NMR spectrum of **5** in DMSO-*d6*. (D) DEPT-135 spectrum of **5** in DMSO-*d6*.

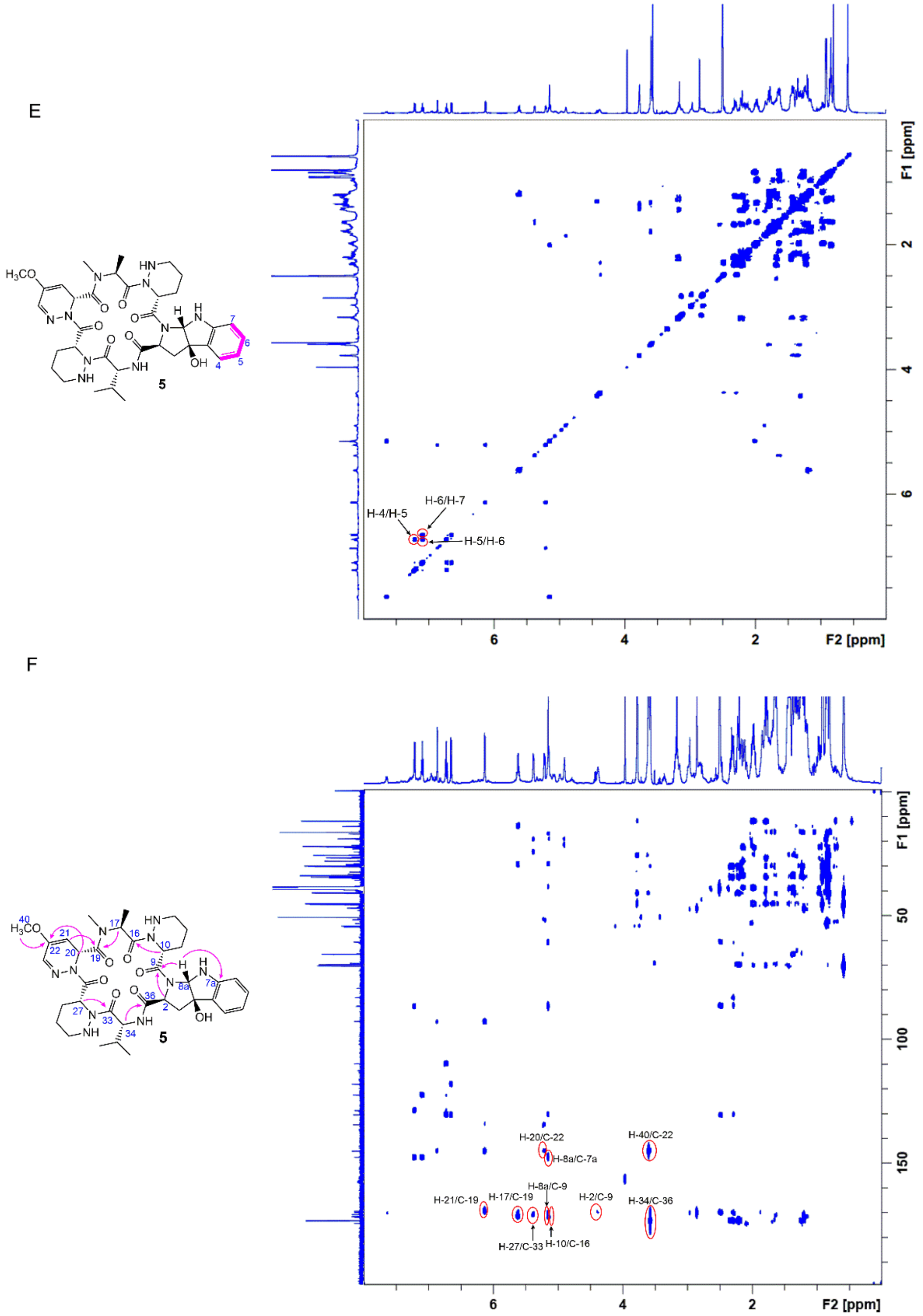

Supplementary Figure S6. Spectral data of **5**. (E) ^{1}H-^{1}H COSY spectrum of **5** in DMSO-$_{d6}$. The key ^{1}H-^{1}H COSY correlation are marked on the structure of **5** with pink bold bonds and at the spectrum with red circles. (F) HMBC spectrum of **5** in DMSO-$_{d6}$. The key HMBC correlations are marked on the structure of **5** with pink arrows and at the spectrum with red circles.

G

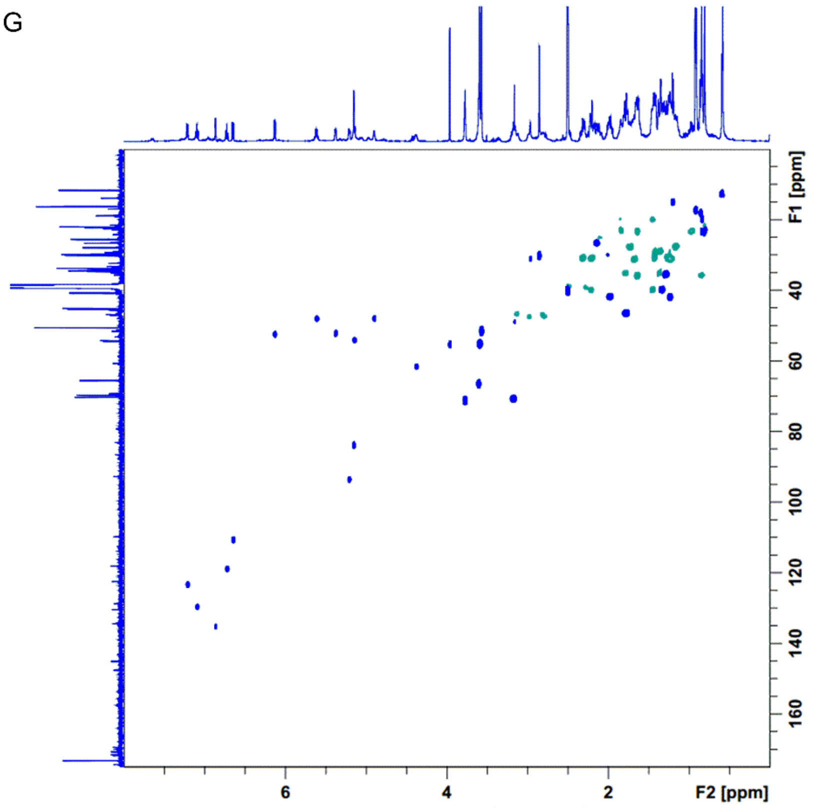

H

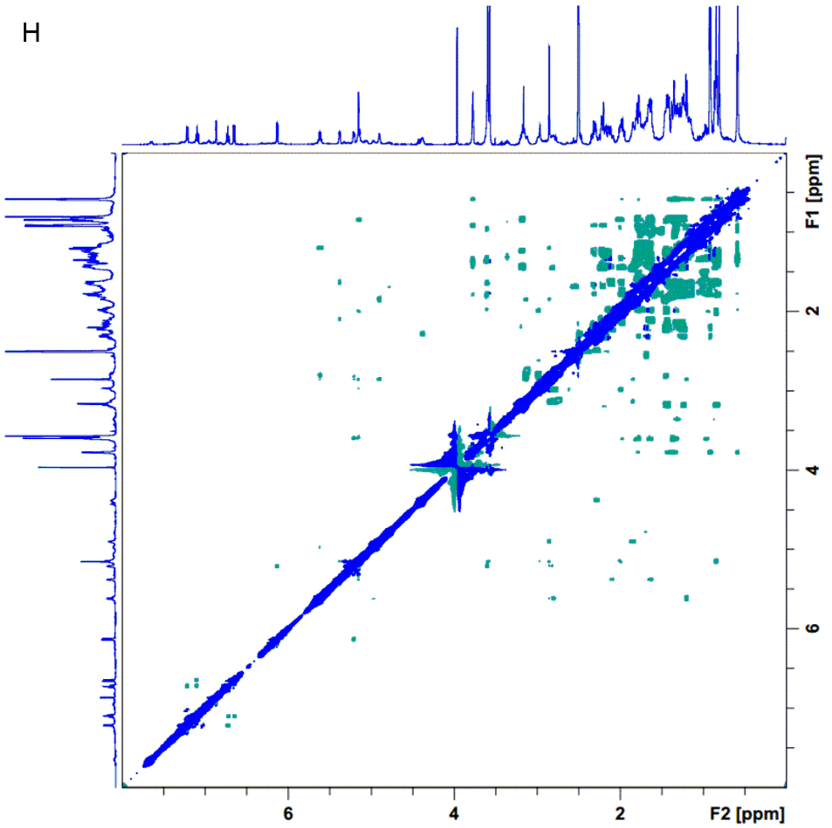

Supplementary Figure S6. Spectral data of **5**. (G) HSQC spectrum of **5** in DMSO-$_{d6}$. (H) ROESY spectrum of **5** in DMSO-$_{d6}$.
